# 3-(1*H*-Imidazol-1-yl)propane­nitrile

**DOI:** 10.1107/S1600536809035685

**Published:** 2009-09-16

**Authors:** Tim Peppel, Martin Köckerling

**Affiliations:** a Universität Rostock, Institut für Chemie, Abteilung Anorganische Chemie/Festkörper­chemie, Albert-Einstein-Strasse 3a, D-18059 Rostock, Germany

## Abstract

The title compound, C_6_H_7_N_3_, has an ethyl­ene group connecting an imidazole ring and a –CN group. These groups are in a staggered conformation. The shortest inter­molecular contact is found between the imidazole N atom and a –CH_2_– group of a neighboring mol­ecule.

## Related literature

For background and applications of ionic liquids, see: Hayashi *et al.* (2006 ▶); Kozlova *et al.* (2009*a* ▶,*b* ▶); Lombardo *et al.* (2007 ▶); Macaev *et al.* (2007 ▶); Sawa & Okamura (1969 ▶); Scheers *et al.* (2008 ▶); Visser *et al.* (2001 ▶); Wang *et al.* (2003 ▶); Wasserscheid & Keim (2000 ▶); Xu *et al.* (2007 ▶); Yamauchi & Masui (1976 ▶); Yang *et al.* (2006 ▶).
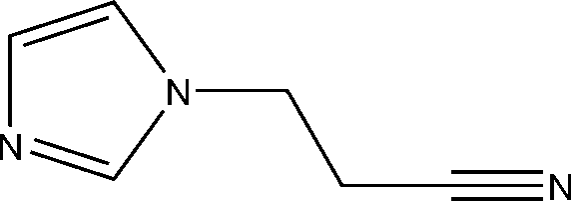


## Experimental

### 

#### Crystal data

C_6_H_7_N_3_
*M* *_r_* = 121.15
Monoclinic, 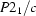

*a* = 7.2712 (3) Å
*b* = 5.5917 (2) Å
*c* = 15.4625 (5) Å
β = 100.979 (1)°
*V* = 617.17 (4) Å^3^
*Z* = 4
Mo *K*α radiation
μ = 0.09 mm^−1^
*T* = 173 K
0.45 × 0.40 × 0.30 mm

#### Data collection

Bruker–Nonius X8 APEX diffractometer
Absorption correction: multi-scan (*SADABS*; Bruker, 2007 ▶) *T*_min_ = 0.946, *T*_max_ = 0.975
11604 measured reflections
1542 independent reflections
1456 reflections with *I* > 2σ(*I*)
*R*_int_ = 0.018

#### Refinement

*R*[*F* ^2^ > 2σ(*F* ^2^)] = 0.032
*wR*(*F* ^2^) = 0.086
*S* = 1.04
1542 reflections
111 parameters
All H-atom parameters refined
Δρ_max_ = 0.27 e Å^−3^
Δρ_min_ = −0.16 e Å^−3^

### 

Data collection: *SMART* (Bruker, 2007 ▶); cell refinement: *SAINT* (Bruker, 2007 ▶); data reduction: *SAINT*; program(s) used to solve structure: *SHELXS97* (Sheldrick, 2008 ▶); program(s) used to refine structure: *SHELXL97* (Sheldrick, 2008 ▶); molecular graphics: *DIAMOND* (Brandenburg, 1999 ▶); software used to prepare material for publication: *ct.exe* (Köckerling, 1996 ▶).

## Supplementary Material

Crystal structure: contains datablocks global, I. DOI: 10.1107/S1600536809035685/om2269sup1.cif

Structure factors: contains datablocks I. DOI: 10.1107/S1600536809035685/om2269Isup2.hkl

Additional supplementary materials:  crystallographic information; 3D view; checkCIF report

## Figures and Tables

**Table 1:** Hydrogen-bond geometry (Å, °)

*D*—H⋯*A*	*D*—H	H⋯*A*	*D*⋯*A*	*D*—H⋯*A*
C5—H5*A*⋯N2^i^	0.97 (1)	2.66 (1)	3.366 (1)	135.7 (9)

Symmetry code: (i) 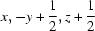.

**Fig. 1.**
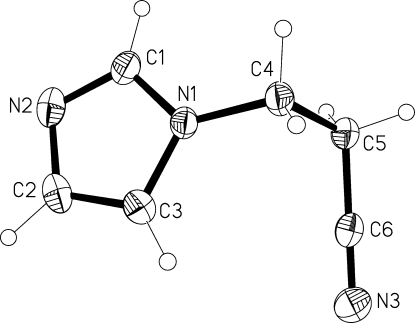
Molecular structure of 3-(1H-imidazol-1-yl)propanenitrile with atom labelling scheme. Displacement ellipsoids are drawn at the 50% probability level.

**Fig. 2.**
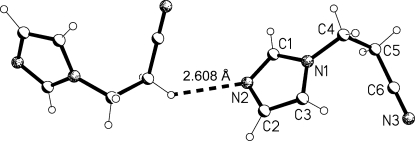
Two neighboring C6H7N3 molecules with the short N2···H(C5) contact.

**Fig. 3.**
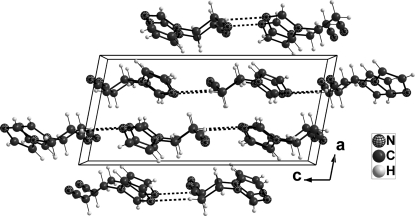
Packing diagram of 3-(1H-imidazol-1-yl)propanenitrile in a view down the crystallographic b direction.

## supplementary crystallographic information

### Comment

Ionic Liquids (ILs) are more and more attracting remarkable attention as new solvents and reaction media for organic and inorganic synthesis and catalysis in order to replace volatile organic solvents (Wasserscheid & Keim, 2000). Imidazolium based ILs have emerged as leading candidates since they have very low vapor pressures, are moisture and air stable, and are highly solvating for both molecular and ionic species. Furthermore, ILs are finding use in separation processes (Visser *et al.*, 2001), in battery applications (Scheers *et al.*, 2008), as electrolytes in solar cells (Wang *et al.*, 2003), or as part of metal catalysts and magnetic liquids (Lombardo *et al.*, 2007; Hayashi *et al.*, 2006; Kozlova *et al.*, 2009a; Kozlova *et al.*, 2009b). Ionic Liquids and imidazole compounds incorporating nitrile functionalities have shown applications in heterocyclic and terpene chemistry (Macaev *et al.*, 2007; Yamauchi & Masui, 1976), in Michael addition or aza-Michael reactions (Xu *et al.*, 2007; Yang *et al.*, 2006).

In this context we present the molecular and single-crystal structure of 3-(1*H*-imidazol-1-yl)propanenitrile, an important precursor of nitrile functionalized ILs. Although all other sources report this compound to be a liquid at room temperature, there is one single indication in the literature that it is a solid melting at 33–35 °C (Sawa & Okamura, 1969). A freshly distilled sample crystallizes spontaneously within days, forming big block-shaped colourless crystals, which are barely deliquescent and melting at 37 °C.

All the bond lengths are within the expected ranges. The molecular structure of the title compound is shown in Figure 1. The C6–N3 bond length of 1.141 (1) Å indicates the triple-bond nitrile character. This nitrile group is connected through an ethylene group with the planar imidazolyl ring. The ethylene group has a staggered conformation with an N1–C4–C5–C6 torsion angle of -65.43 (9)°.

The shortest intermolecular contacts are found between one of the H atoms bonded at C5 and the N2 atom of the symmetry equivalent neighboring molecule. The H5A···N2^#^ distance measures 2.66 (1) Å, the corresponding C5···N2^#^ distance 3.366 (1) Å, and the C5–H5a···N2^#^ angle 135.6° (symmetry code #: x, 1/2-y, z+1/2). Therefore the contact can be considered as a weak hydrogen bond. Figure 2 shows the orientation of two molecules, which are attached through this weak hydrogen bond.

In the crystal the molecules are arranged in rows, such that this weak hydrogen bond is oriented approximately parallel to the crystallographic c direction. Due to the c glide plane, every second row, stacked along a is shifted by c/2. The packing diagram, Figure 3, shows this assembly.

### Experimental

1*H*-Imidazole (50.0 g, 0.7 mol) and acrylonitrile (117.0 g, 2.2 mol) were refluxed in 150 ml of ethanol overnight. Excess acrylonitrile and the solvent were evaporated and the residue was distilled in vacuum, yielding a colourless supercooled liquid, which crystallized at room temperature after several days. Yield: 75.0 g (84%), mp 37 °C.

### Refinement

The positions of the hydrogen atoms were located from a difference Fourier map and refined isotropically.

### Crystal data

**Table:**

C_6_H_7_N_3_	*D*_x_ = 1.304 Mg m^−^^3^
*M**_r_* = 121.15	Melting point: 310 K
Monoclinic, *P*2_1_/*c*	Mo *K*α radiation, λ = 0.71073 Å
*a* = 7.2712 (3) Å	Cell parameters from 8906 reflections
*b* = 5.5917 (2) Å	θ = 2.7–28.3°
*c* = 15.4625 (5) Å	µ = 0.09 mm^−^^1^
β = 100.979 (1)°	*T* = 173 K
*V* = 617.17 (4) Å^3^	Block, colourless
*Z* = 4	0.45 × 0.40 × 0.30 mm
*F*(000) = 256	

### Data collection

**Table:**

Bruker–Nonius X8 Apex diffractometer	1542 independent reflections
Radiation source: fine-focus sealed tube	1456 reflections with *I* > 2σ(*I*)
graphite	*R*_int_ = 0.018
φ and ω scans	θ_max_ = 28.3°, θ_min_ = 3.5°
Absorption correction: multi-scan (*SADABS*; Bruker, 2007)	*h* = −9→9
*T*_min_ = 0.946, *T*_max_ = 0.975	*k* = −7→7
11604 measured reflections	*l* = −20→16

### Refinement

**Table:**

Refinement on *F*^2^	Secondary atom site location: difference Fourier map
Least-squares matrix: full	Hydrogen site location: inferred from neighbouring sites
*R*[*F*^2^ > 2σ(*F*^2^)] = 0.032	All H-atom parameters refined
*wR*(*F*^2^) = 0.086	*w* = 1/[σ^2^(*F*_o_^2^) + (0.0486*P*)^2^ + 0.1206*P*] where *P* = (*F*_o_^2^ + 2*F*_c_^2^)/3
*S* = 1.04	(Δ/σ)_max_ < 0.001
1542 reflections	Δρ_max_ = 0.27 e Å^−^^3^
111 parameters	Δρ_min_ = −0.16 e Å^−^^3^
0 restraints	Extinction correction: *SHELXL97* (Sheldrick, 2008), Fc^*^=kFc[1+0.001xFc^2^λ^3^/sin(2θ)]^-1/4^
Primary atom site location: structure-invariant direct methods	Extinction coefficient: 0.064 (15)

### Special details

**Table:**

Geometry. All e.s.d.'s (except the e.s.d. in the dihedral angle between two l.s. planes) are estimated using the full covariance matrix. The cell e.s.d.'s are taken into account individually in the estimation of e.s.d.'s in distances, angles and torsion angles; correlations between e.s.d.'s in cell parameters are only used when they are defined by crystal symmetry. An approximate (isotropic) treatment of cell e.s.d.'s is used for estimating e.s.d.'s involving l.s. planes.
Refinement. Refinement of *F*^2^ against ALL reflections. The weighted *R*-factor *wR* and goodness of fit *S* are based on *F*^2^, conventional *R*-factors *R* are based on *F*, with *F* set to zero for negative *F*^2^. The threshold expression of *F*^2^ > σ(*F*^2^) is used only for calculating *R*-factors(gt) *etc*. and is not relevant to the choice of reflections for refinement. *R*-factors based on *F*^2^ are statistically about twice as large as those based on *F*, and *R*- factors based on ALL data will be even larger.

### Fractional atomic coordinates and isotropic or equivalent isotropic displacement parameters (Å^2^)

**Table:**

	*x*	*y*	*z*	*U*_iso_*/*U*_eq_	
N1	0.76561 (9)	0.2518 (1)	0.30688 (4)	0.0207 (2)	
C1	0.6541 (1)	0.1964 (2)	0.22892 (5)	0.0254 (2)	
H1	0.579 (2)	0.053 (2)	0.2217 (7)	0.033 (3)*	
N2	0.6623 (1)	0.3580 (1)	0.16800 (4)	0.0302 (2)	
C2	0.7866 (1)	0.5266 (2)	0.20919 (6)	0.0281 (2)	
H2	0.817 (2)	0.670 (2)	0.1771 (8)	0.037 (3)*	
C3	0.8519 (1)	0.4648 (1)	0.29465 (5)	0.0249 (2)	
H3	0.944 (2)	0.535 (2)	0.3408 (8)	0.035 (3)*	
C4	0.7876 (1)	0.1115 (1)	0.38757 (5)	0.0239 (2)	
H4A	0.919 (2)	0.109 (2)	0.4147 (7)	0.030 (3)*	
H4B	0.744 (2)	−0.051 (2)	0.3700 (7)	0.031 (3)*	
C5	0.6708 (1)	0.2069 (2)	0.45232 (5)	0.0254 (2)	
H5A	0.683 (2)	0.100 (2)	0.5022 (8)	0.037 (3)*	
H5B	0.538 (2)	0.217 (2)	0.4243 (7)	0.035 (3)*	
C6	0.7298 (1)	0.4436 (2)	0.48692 (5)	0.0244 (2)	
N3	0.7779 (1)	0.6275 (1)	0.51425 (5)	0.0333 (2)	

### Atomic displacement parameters (Å^2^)

**Table:**

	*U*^11^	*U*^22^	*U*^33^	*U*^12^	*U*^13^	*U*^23^
N1	0.0242 (3)	0.0212 (3)	0.0176 (3)	0.0012 (2)	0.0061 (2)	0.0007 (2)
C1	0.0293 (4)	0.0279 (4)	0.0194 (4)	0.0001 (3)	0.0057 (3)	−0.0026 (3)
N2	0.0345 (4)	0.0371 (4)	0.0196 (3)	0.0033 (3)	0.0064 (3)	0.0029 (3)
C2	0.0290 (4)	0.0307 (4)	0.0271 (4)	0.0027 (3)	0.0113 (3)	0.0075 (3)
C3	0.0246 (4)	0.0249 (4)	0.0257 (4)	−0.0015 (3)	0.0065 (3)	0.0023 (3)
C4	0.0318 (4)	0.0213 (4)	0.0188 (3)	0.0026 (3)	0.0058 (3)	0.0025 (3)
C5	0.0321 (4)	0.0265 (4)	0.0190 (4)	−0.0056 (3)	0.0084 (3)	−0.0011 (3)
C6	0.0265 (4)	0.0288 (4)	0.0186 (3)	0.0011 (3)	0.0060 (3)	−0.0002 (3)
N3	0.0386 (4)	0.0303 (4)	0.0313 (4)	−0.0002 (3)	0.0078 (3)	−0.0047 (3)

### Geometric parameters (Å, °)

**Table:**

N1—C1	1.354 (1)	C6—N3	1.141 (1)
N1—C3	1.376 (1)	C1—H1	0.97 (1)
N1—C4	1.4565 (9)	C2—H2	0.99 (1)
C1—N2	1.315 (1)	C3—H3	0.97 (1)
N2—C2	1.376 (1)	C4—H4A	0.97 (1)
C2—C3	1.361 (1)	C4—H4B	0.99 (1)
C4—C5	1.527 (1)	C5—H5A	0.97 (1)
C5—C6	1.461 (1)	C5—H5B	0.98 (1)
			
C1—N1—C3	106.68 (6)	H2—C2—N2	120.9 (7)
C1—N1—C4	126.06 (7)	H3—C3—C2	132.8 (7)
C3—N1—C4	127.27 (7)	H3—C3—N1	121.4 (7)
N2—C1—N1	112.30 (7)	H4A—C4—H4B	110.4 (9)
C1—N2—C2	104.76 (7)	H4A—C4—N1	108.5 (7)
C3—C2—N2	110.64 (7)	H4B—C4—N1	106.5 (7)
C2—C3—N1	105.63 (7)	H4A—C4—C5	110.2 (7)
N1—C4—C5	112.92 (6)	H4B—C4—C5	108.2 (7)
C6—C5—C4	113.28 (7)	H5A—C5—H5B	109 (1)
N3—C6—C5	179.24 (9)	H5A—C5—C6	107.0 (7)
H1—C1—N2	126.2 (7)	H5B—C5—C6	107.9 (7)
H1—C1—N1	121.5 (7)	H5A—C5—C4	109.2 (7)
H2—C2—C3	128.4 (7)	H5B—C5—C4	110.7 (7)

### Hydrogen-bond geometry (Å, °)

**Table:**

*D*—H···*A*	*D*—H	H···*A*	*D*···*A*	*D*—H···*A*
C5—H5A···N2^i^	0.97 (1)	2.66 (1)	3.366 (1)	135.7 (9)

Symmetry codes: (i) *x*, −*y*+1/2, *z*+1/2.
